# How to Scale Up a Lifesaving Molecule in a Matter of Months

**DOI:** 10.1021/acscentsci.3c01089

**Published:** 2023-09-05

**Authors:** Bethany Halford

On July 22, 2020, medicinal chemists at the drugmaker Pfizer made a molecule that they called PF-07321332, 1 of about 20 compounds they prepared that day. The scientists were searching for a way to shut down SARS-CoV-2, the virus that causes COVID-19—a disease that was responsible for more than 25 000 deaths in the U.S. alone that same month.

The researchers did not know it at the time, but their discovery of PF-07321332 started a clock ticking. Over the next few months, scientists at the company discovered that the molecule was a powerful inhibitor of SARS-CoV-2’s main protease (also known as the 3CL protease) and had the right mix of properties to be taken as a pill. They eventually renamed it nirmatrelvir, and the race was on to make enough to treat millions of people with COVID-19.

**Figure:**
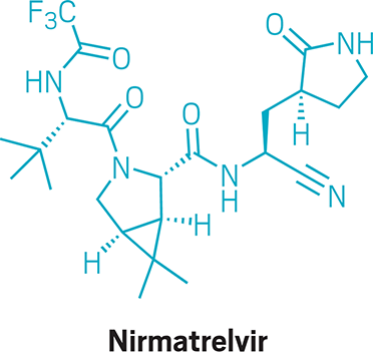
Credit: C&EN

**Figure:**
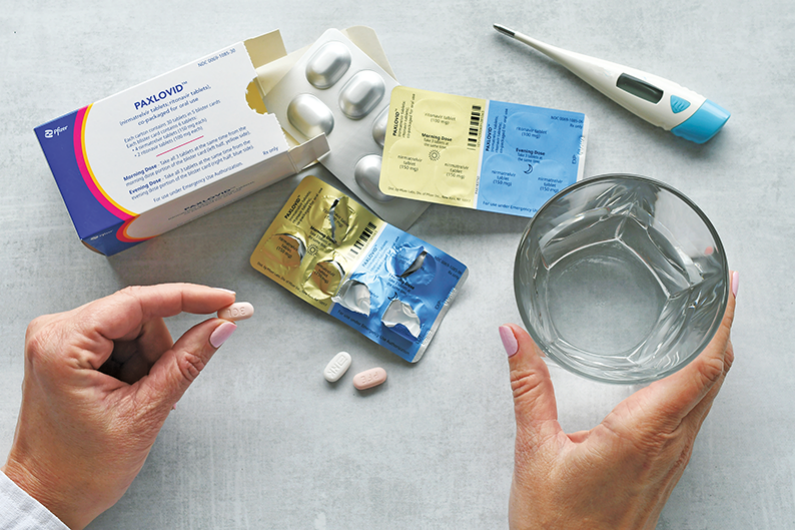
Millions of prescriptions for Paxlovid have been written since the US Food and Drug Administration authorized it for emergency use in December 2021. Credit: Shutterstock

Just 17 months after nirmatrelvir’s discovery, the compound was heading to patients. In December 2021, the U.S. Food and Drug Administration gave an emergency use authorization for the antiviral Paxlovid to treat COVID-19.

Paxlovid is a combination of nirmatrelvir and ritonavir, which boosts nirmatrelvir’s circulation time in the body. Pfizer provided the U.S. government with 20 million courses of Paxlovid in 2022. It has saved countless lives. A recent study also suggests it might prevent people from developing long COVID. This month the FDA is scheduled to decide whether Paxlovid should have full status as a drug.

To go from just milligrams of nirmatrelvir to enough to supply millions of people so quickly required an intensive effort from Pfizer’s process chemistry team.

Process chemists figure out how to make kilograms or even metric tons of a molecule that has been made only on the gram scale in a research lab. Their task typically takes years as a drug candidate moves through preclinical and clinical trials and regulatory review. Going from the first laboratory synthesis to an emergency use authorization in 17 months is a speed record for Pfizer and possibly for the entire pharmaceutical industry.

R. Matt Weekly, who led Pfizer’s process chemistry team for nirmatrelvir, says he and his colleagues knew that whatever molecule came out of the medicinal chemistry campaign, they were going to have to figure out how to make a lot of it quickly. “We were preparing to treat a world population,” he says.

“There was a period of time where the vast majority of R&D efforts at Pfizer—between the vaccine and the oral treatment—it was all focused on COVID,” says John A. Ragan, a 30-year process chemistry veteran at Pfizer. He worked on the nirmatrelvir scale-up before retiring in April.

Pfizer’s process chemists set aside their other commitments and put all their effort into scaling up nirmatrelvir so there’d be enough of it if the FDA gave it the emergency use authorization, Ragan says. “Everybody was all in on this program, and everything else was on hold.”

The company decided to devote significant resources to the scale-up of nirmatrelvir not long after it showed promise in preclinical studies. “We basically said, ‘Look, we’re going to assume that this works. We’re going to assume success and make investments and business decisions at financial risk,’ ” Ragan says.

**Figure:**
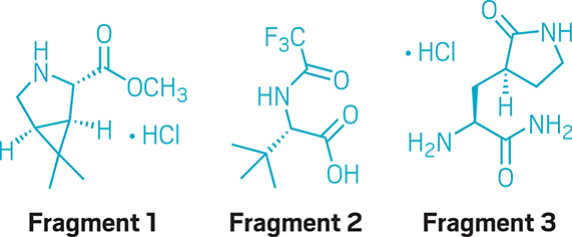
Credit: C&EN

Pfizer began purchasing large quantities of starting materials for the synthesis before executives were sure that nirmatrelvir would cross all the safety and regulatory hurdles. In total, Pfizer spokesperson Kit Longley tells C&EN the company invested nearly $1.5 billion to support the development and manufacture of Paxlovid before its emergency use authorization.

The Pfizer process chemistry team outlines its strategy for the nirmatrelvir scale-up in a recent *ACS Central Science* paper. The group needed more than 100 t of reagents, and more than 100 people worked on the project across approximately 20 R&D sites, all during a pandemic.

“Having our employees so dedicated and committed to what they were doing, to put in all those hours when everybody else was on lockdown, it was a challenge,” Weekly says. “But to me, it was also remarkable—the commitment and dedication that we saw out of our colleagues.”

To synthesize nirmatrelvir, the chemists broke the molecule into three fundamental building blocks. Fragment 1, a bicyclic pyrrolidine, had been used in the synthesis of boceprevir, a treatment for hepatitis C virus developed by Schering-Plough and Merck & Co. Merck withdrew boceprevir from the market in 2015 because better treatments for hepatitis C virus were available, but Ragan says suppliers of the bicyclic pyrrolidine were able to restart their processes for making the molecule.

Fragment 2’s synthesis was straightforward. It was made by adding a trifluoroacetyl group to commercially available tertiary leucine.

Fragment 3, a primary amide, had been part of a clinical candidate that Pfizer chemists in La Jolla, California made back in 2001, and it was a component in several subsequent clinical candidates that never became drugs. Because of that “there were pretty good synthetic routes available to that compound,” Ragan says. He points out that the work done more than 20 years ago on that fragment paid off, even though it did not appear in a commercial drug at the time.

The chemists ran into a problem when coupling fragments 1 and 2. They had figured out a way to streamline the discovery team’s route by making a lithium salt of fragment 1. But when they tried to make the lithium salt on scales over 100 kg, the compound formed hairlike needles that coalesced into thick slurries that mucked up their reactors. This problem meant “we actually created an additional team to do nothing but take a look at potential replacements for the lithium salt,” Weekly says. The chemists eventually found that the sodium salt was the best option.

Ragan says that nirmatrelvir is not the most challenging molecule to make from a synthetic standpoint. The biggest challenge in this project, he says, was working out the supply chain logistics. “We had never tried to do something this quickly and this aggressively,” he says. Working with contract manufacturing organizations and suppliers was key. For example, the company found five to seven suppliers for each starting material needed for the synthesis. Typically, drugmakers use just two suppliers for their starting materials.

That Pfizer was able to develop a commercial manufacturing process in 17 months is “an unbelievable feat,” says Kai Rossen, editor in chief of *Organic Process Research and Development*. “It shows that they clearly are capable, organized, have the resources and the willingness to make gutsy decisions to move forward and do the whole development in a time frame that would have been considered impossible,” he says. *Organic Process Research and Development* is published by the American Chemical Society, which also publishes Chemical & Engineering News and *ACS Central Science*.

Rossen says it would have been tough for a smaller company to accomplish the scale-up on such a short timeline. “Only a top 10 pharma company has the resources, the know-how, the network, and the understanding to do that,” he says.

Rossen also points to impressively quick scale-ups of two other COVID-19 antivirals: Merck’s molnupiravir and Shionogi’s ensitrelvir.

What the Pfizer team accomplished in such a short period is remarkable. Ragan says the company’s chemists showed such achievements are possible with sufficient resources and the willingness to take on risk. But, he adds, “it’s important that leadership understand that we can’t do it for everything.” This is a feat to be taken on only in the face of a global pandemic.

## Bethany Halford is a senior correspondent at

Chemical & Engineering News*, the independent news outlet of the American Chemical Society.*A version of this story appeared in Chemical and Engineering News.

